# The Influence of Fluids With Varying Rheological Properties on the Field of Fluidic Effect During Vitrectomy

**DOI:** 10.1167/tvst.14.8.26

**Published:** 2025-08-20

**Authors:** Daria Vedeniapina, David H. Steel, Richard D. Whalley

**Affiliations:** 1 School of Engineering, Newcastle University, Newcastle upon Tyne, UK; 2 Biosciences Institute, Newcastle University, Newcastle upon Tyne, UK; 3 School of Mechanical & Aerospace Engineering, Queen's University Belfast, Belfast, UK

**Keywords:** vitrectomy, field of effect (FOE), artificial vitreous solution (AVS), rheology, particle image velocimetry (PIV)

## Abstract

**Purpose:**

The purpose of this study was to investigate the field of fluidic effect (FOE) at the vitrectomy port in a range of artificial vitreous solutions (AVS) with varying rheological characteristics to add insight into the effects surgeons observe during vitrectomy and how they change with a variety of console settings.

**Methods:**

Laboratory-based investigation using in vitro testing was carried out at Newcastle University, United Kingdom. Testing was conducted using an Eva Nexus Vitrectomy system (D.O.R.C. Dutch Ophthalmic Research Center [International] B.V., Zuidland, The Netherlands) using dual cutting action vitrectomy probes in 3 gauge sizes (23G, 25G, and 27G). Using AVS, particle image velocimetry (PIV) measurements were conducted for various console settings and AVS concentrations.

**Results:**

Rheological analysis of AVS with varying hyaluronic acid concentrations showed shear thinning and viscoelastic properties, resembling the liquid component of human vitreous humour. PIV during vitrectomy revealed that a dual cutting probe created a focused, elongated jet stream in AVS, contrasting with the symmetrical flow in balanced salt solution (BSS). Key findings included peak fluid velocity magnitude in AVS was 0.5 to 1 mm from the port, broadening with increased vacuum, higher vacuum led to inferior jet deviation, cut rates increased peak velocities, with viscoelasticity influencing fluid flow dynamics, and increasing hyaluronic acid concentration transitioned flow from BSS-like to elongated jet profiles.

**Conclusions:**

Using an AVS with similar rheological properties to the liquid component of human vitreous and a high-speed PIV technique, we evaluated the FOE around vitrectomy probes with a range of surgical relevant variables. Further research into the influence of different vitreous substitutes and surgical parameters is crucial to enhance our understanding and refine vitrectomy procedures.

**Translational Relevance:**

Our findings provide valuable insights into the fluid dynamics during vitrectomy, helping to optimize surgical techniques and improve patient outcomes.

## Introduction

Vitrectomy is being used to treat an expanding range of sight-affecting conditions with a reduced threshold for surgery as technology and safety have improved. Although reducing vitrectomy gauge, faster cut rates, and dual action cutting have driven this trend by facilitating surgery and enhanced intraocular stability, the fluidic effects during vitrectomy are still unclear. We have recently presented a detailed investigation of the field of fluidic effect (FOE) that vitrectomy probes induce in a balanced salt solution (BSS).^1^ We have shown that two symmetrical fluidic zones centered at right angles to the probe port can be observed with a relatively sharp border of high acceleration demarcating a small high flow zone from a larger and more variable low flow zone. This has significant translational importance in explaining the effect of vitrectomy probes as they are brought toward the vitreous and retina. The FOE in vitreous and vitreous-like mimetics has had little study. To date, investigations have concentrated on the changes in bulk flow and fluctuation during aspiration^2^^–^^9^ as compared to BSS. Vitreous is a hyaluronic acid/collagen gel with viscoelastic behavior, which by nature will affect the FOE's characteristics. Human vitreous is highly heterogeneous, changing by age, refraction, and disease type and varying between different parts of the vitreous cavity.^10^^,^^11^ Understanding the FOE around a vitrectomy probe in these various vitreous states is important to surgeons to allow them to predict changes in surgical behavior between cases and as the vitrectomy proceeds. Egg albumen^2^ and porcine vitreous^3^ have been used as human vitreous substitutes, but both differ significantly from human vitreous and are themselves heterogeneous between batches. Artificially produced vitreous substitutes have also been used^4^ and have several advantages compared with naturally occurring substances. They can be formulated to match both the elastic and viscous properties of the liquid component of human vitreous and their properties are consistent and can be measured, allowing viscoelastic effects to be explored and measured. We set out to investigate the FOE at the vitrectomy port in a range of artificial vitreous substitutes (AVS) with varying viscoelasticity to add insight into the effects surgeons observe during vitrectomy and how they change with a variety of console settings.

## Methods

### Experimental Setup

**Figure 1.**
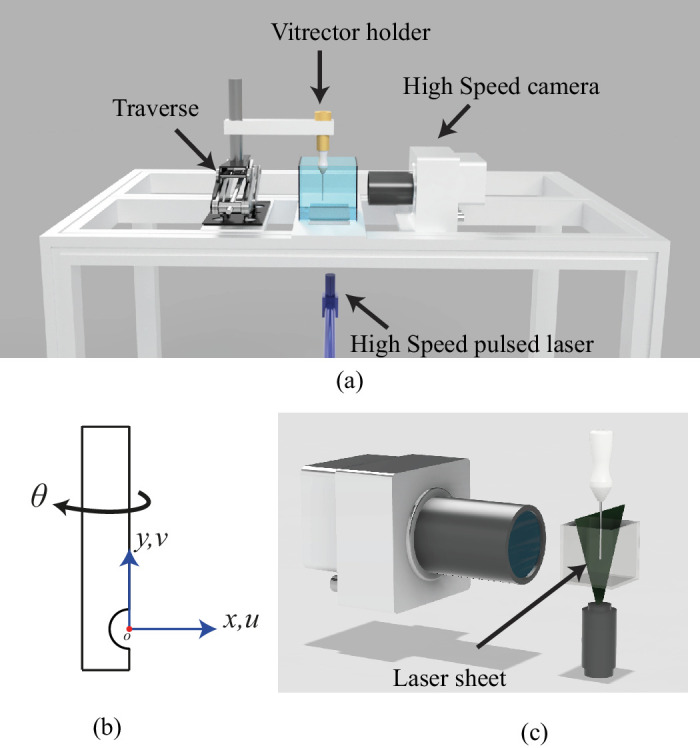
(**a**) Schematic of the experimental setup, and (**b**) cutter schematic and the coordinate system used in the paper. *θ* = 0 is for the present orientation, which increases in the direction of the arrow, (**c**) zoomed-in view of the high-speed pulsed laser and camera.

To investigate the FOE of the vitreous cutters, an in vitro experimental setup was designed at the Fluid Dynamics Laboratory, Newcastle University, United Kingdom. We used the Eva Nexus Vitrectomy system (D.O.R.C. Dutch Ophthalmic Research Center [International] B.V.) with 23G, 25G, and 27G dual action cutting probes. The cutter was secured in a custom holder that could rotate in 1 degree increments, allowing precise alignment of its port with the particle image velocimetry (PIV) laser-sheet plane. That holder sat on a 3-axis manual stage: the x and z axes move in 0.1 mm steps, and the y axis in 1 mm steps. These resolutions let us position the cutter port exactly at the PIV camera's focal plane and adjust its depth in the tank with sub-millimeter accuracy. A photograph of the complete setup (cutter, XYZ stage, and PIV tank) is shown in Supplementary Figure S1. See Figure 1 for a schematic representation of the setup. The cutter tip was immersed (25 mm depth) in a transparent open container with l × w × h of 35 × 35 × 55 mm filled with AVS.

### Preparation and Characterization of AVS

We conducted experiments on AVS composed of hyaluronic acid (HA) sodium salt (Streptococcus equi 91%; Thermo Scientific) and deionized water (Thermo Scientific). We varied HA concentrations from 0.5 to 4 milligrams per milliliter (mg/mL) according to Nepita et al.^12^ The preparation consisted of mixing sodium salt of HA with deionized water under constant mixing on a hotplate magnetic stirrer (Fisherbrand AREX 5 Hotplate Stirrer) and a 30-minute heating period up to 100°C for full dissolution of the components. After this the mixture was sealed with cling film and left to settle for 24 hours. We tested four different AVS (referred to herein as HA-0.5 to 4, based on the HA concentration) to replicate the liquid component of human vitreous, comparing rheological data and flow rates under various vacuums to simulate surgical scenarios.

The rheological characterization, including complex viscosity (*η*∗), loss modulus (G”), and storage modulus (G’), was performed using an Anton Paar MCR302e rheometer. Data were analyzed with RheoCompass and Matlab. The rheometer, equipped with a high-resolution optical encoder and an air bearing system, had a torque resolution of 1 nN/m within a range of 0.5 µN/m to 230 mN/m, and angular deflection resolution of 0.05 µrad.

We performed small-amplitude oscillatory shear tests at frequencies from 0.01 to 1 hertz (Hz) with a shear strain of 0.5%, ensuring measurements were within the linear viscoelastic region. Measurements were stopped after 45 minutes. All experiments used a 50 mm diameter parallel plate geometry. For a range of experiments a 50 mm diameter, 2 degrees stainless steel cone was used, however, there were no significant differences between measurements with the 2 geometries at the range of shear rates tested. For the cone-and-plate and parallel plate geometries at high shear rates, the same values of total uncertainty apply because in that range the uncertainty is dominated by the angular speed contribution which is independent of the geometry used.^13^ The temperature of each sample was held constant at 20°C ± 0.1°C by a Peltier plate. Each measurement was repeated three times. All results were compared to the liquid component of human vitreous.^14^ To ensure the sample did not degrade with time, measurements of shear viscosity, storage, and loss modulus were conducted on HA-2 over a 5-day period. All measurements of shear viscosity, storage, and loss modulus were within 5% from an average of 15 (3 per day) independent measurements per sample as can be seen from Supplementary Figure S2. No sample was used after a 5-day period. All samples when not in use were stored at 4°C.

### Particle Image Velocimetry 

The flow field around the cutter was assessed using PIV. The PIV system included a Phantom VEO-E 310L high-speed camera (1280 × 800 pixels at full resolution) and a Litron double-pulsed Nd-YLF laser (527 nm, 15 mJ/pulse at 1 kilohertz [kHz]). The laser sheet (0.5 mm thick) was directed from underneath the cutter to avoid shadows. AVS was seeded with 8 µm neutrally buoyant red polystyrene fluorescent particles (1050 kg/m*) with an absorption/emission wavelength of 542/612 nm. An optical band-pass filter (600 ± 50 nm) was used to remove unwanted reflections. The Stokes number (*S_k_* = particle response time/characteristic time scale of the flow^15^) for the seeding particles was 4.15 × 10*^−^*^8^ ≤ S*_k_* ≤ 4*.*84 × 10*^−^*^5^ depending on the aspiration conditions, meeting the acceptable limit (*S_k_ <* 0.1) for accurate flow tracing.^16^ Particle concentration was 0.2 mg/mL.^17^

To prevent particle aggregation, 0.125% Polysorbate 20 (Fisher BioReagents) was added without altering the viscosity. The dosing was done in the container after mixing. The flow field was captured with a 60 mm lens over a 5.5 × 5 mm field of view. For each measurement, 2000 image pairs were acquired at 2 to 5 kHz. To achieve the desired acquisition frequency, the camera resolution was reduced to 832 × 760 pixels above 2 kHz.

The acquired images were processed using LaVision's Davis software using a multi-pass cross-correlation scheme with a final interrogation area of 32 × 32 pixels with an overlap of 75%, yielding 95 × 104 velocity vectors with a spatial resolution of 0.053 mm. Each experiment was repeated three times. The experiments across gauge, vacuum, AVS concentration, and cut rate are summarized in the Table. The flow in AVS due to the vitreous cutter can be described as a jet flow into the cutter because fluid surrounding the cutter is drawn into the port. Therefore, the origin of the coordinate system was taken at the port, and the axes defined as in Figure 1. According to the coordinate system the aspirated flow is directed along the negative “-x,” both negative and positive “y” directions depending on the case assessed. To aid interpretation, the velocity moving into the cutter along the x-direction is represented as a positive value throughout the paper. The instantaneous velocities in the horizontal and vertical directions are denoted by u and v, respectively. The time-averaged velocities in the horizontal and vertical directions are represented by *u* and *v*, respectively. The time-averaged velocity magnitude is denoted as

$$\begin{array}{c} \bar{U}=\sqrt{\left(\bar{u^{2}+v^{2}}\right)}. \end{array}$$

**Table.** Experimental Conditions Tested With PIV

Test	23 G	25 G	27 G
Effect of gauge	650 mm Hg	650 mm Hg	650 mm Hg
	16,000	16,000	16,000
	CPM	CPM	CPM
	HA-2	HA-2	HA-2
Effect of vacuum	X	200, 400, 550, and 650	X
		Mm Hg 16,000 CPM	
		HA-2	
Effect of concentration	650 mm Hg	X	X
	16,000		
	CPM HA-1, -1.5, -		
	2, -3, and -4		
Effect of cut rate	X	650 mm Hg	X
		0–16,000	
		CPM	
		HA-2	

The X denotes an untested parameter.

### Volume Flow Rate Measurements

The 23 G, 25 G, and the 27 G dual-blade cutters driven by the Eva Nexus vitrectomy machine (DORC Ltd.) were studied. Cut rates from 2000 to 16,000 cuts per minute (CPM) were chosen to cover clinically relevant cut rates and match prior studies.^3^^,^^6^^,^^9^^,^^12^ To evaluate the effect of vacuum and flow rates we used a cut rate of 16,000 CPM as the EVA Nexus TDC cutter's technical limit, the setting most surgeons use and to minimize frequency-dependent effects and traction while keeping flow stable and isolating vacuum-dependent trends.^2^^–^^4^^,^^6^^,^^12^^,^^18^ All vacuum- and cut-rate experiments were done with a 25 G cutter, based on it being the commonest gauge used clinically and allowing direct comparison with previous bench and clinical studies. For AVS concentration experiments, we switched to the larger lumen 23 G cutter to avoid gauge-related flow limitations and focus on viscosity effects.

To measure average aspiration rates, a 100 mL beaker was filled with approximately 64 mg of AVS and placed on a sensitive mass balance (KERN PCB100-3, resolution 0.001 g, 3 seconds of stabilization time). The vitreous cutter was held vertically by the arms of a support stand, and the tip of the cutter was submerged 30 mm into the AVS. The mass of AVS aspirated from the beaker was measured after 30 seconds and each measurement was repeated 3 times.^19^ The volume of the AVS aspirated by the 23 G, 25 G, and 27 G cutters was calculated from the reduction of the mass with a range of set cut rates. Flow was set in the vacuum controlled mode at aspiration pressures of 200, 300, 400, 500, 550, 600, and 650 millimeters of mercury (mm Hg). To calculate the aspiration flow rates, the aspirated mass was divided by the density of the AVS, which was found to be constant at 0.972 mg/L via a calibrated cylinder. The results were compared with our previously published rates in BSS.

### Characterization of the FOE in AVS

A measure of the jet thickness (*δ*) is a half–value width, that is, the local distance of the points with half the maximum velocity as a function of downstream distance.^16^ In brief, the method involves analyzing the velocity distribution along several vertical slices of the jet, identifying points where the velocity drops to a certain fraction (50%) of the maximum, and using the distance between these points to determine the jet's thickness. This process is repeated at various positions along the jet, and the results are plotted to show how the jet thickness changes with distance. The method for finding the length of the jet (*L*) involves identifying where the jet's width is half of its maximum value as one moves along the jet's axis. This length is measured as the distance from the cutter port to the position where the jet first reaches this half-width condition. This approach allows us to quantify the extent of the jet before it thins to a critical threshold.^16^ The jet angle (*θ*) is identified by how many degrees the centerline of the jet has deviated from the *y* = 0 line of the time-averaged velocity field.

## Results

### Rheological Behavior of Different AVS

**Figure 2.**
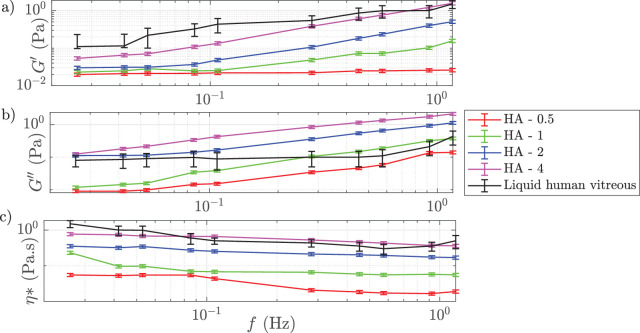
Rheological properties of AVS at 25°C. Small-amplitude oscillatory shear tests: (**a**) Storage modulus G’ and (**b**) loss modulus G” (**c**) complex viscosity *η*∗ as a function of frequency. The liquid component of human vitreous data (*black line*) is a mean of 39 independent samples (aged 62 ± 15 years), uncertainty bars represent 95% confidence intervals.^14^ Uncertainty bars on our samples show the standard deviation of three independent measurements.

**Figure 3.**
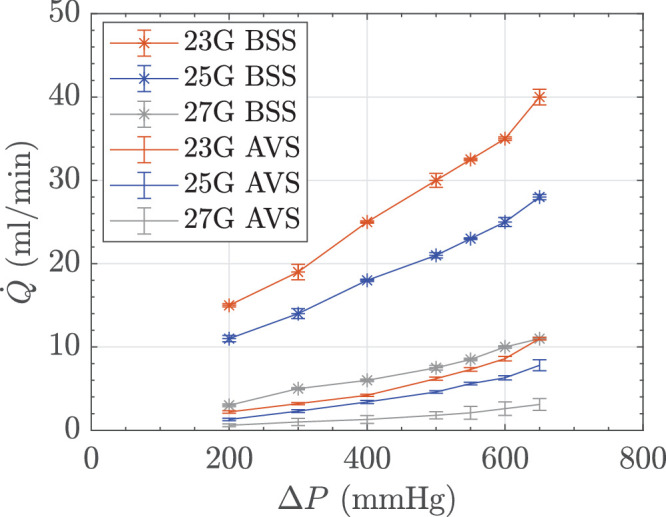
Variation in flow rate ($\dot{Q}$) with applied vacuum (∆*P*) at 16,000 CPM. Uncertainty bars show the standard deviation of three independent measurements.

The storage and loss modulus data and complex viscosity of the tested AVSs is shown in Figure 2. As the rotational frequency of the rheometer increased, the viscosity of the AVSs decreased, indicating shear thinning properties with a viscosity ranging from approximately 0.03 Pascal (Pa) to 0.80 Pa at lower frequencies and converging to lower values at higher shear rates. The storage modulus increased with frequency, reflecting the elastic nature of the solutions, with values ranging from near 0 to approximately 2 Pa. Similarly, the loss modulus increased with frequency, with values reaching up to 2.50 Pa, demonstrating a frequency-dependent viscoelastic response. As the frequency increases, the AVSs are subject to a progressively greater shear stress, which breaks the fluids microstructure (the physical crosslinking points between polymer chains), thus facilitating fluid motion.^20^ AVS HA-2 and HA-4 most closely matched the rheological properties of the liquid component of human vitreous and HA-2 was chosen for most of the tests performed because the achievable flow rates closely matched testing using human vitreous in previous studies. This is also in agreement with the measurements performed by Nepita et al. (see Figs. 3a, 3b, Supplementary Figs. S3, S4).^12^

### Bulk Flow Rates in AVS and BSS

Figure 3 illustrates the relationship between vacuum and flow rate for HA-2 and BSS at 16,000 CPM. In both media, there was an increase in flow rate with higher vacuum. Flow rates in BSS were higher and exhibited a greater rise with increasing vacuum, reaching maximum rates approximately 4 times higher than HA-2.

#### Velocity Fields in AVS and BSS With Matched Flow Rates

**Figure 4.**
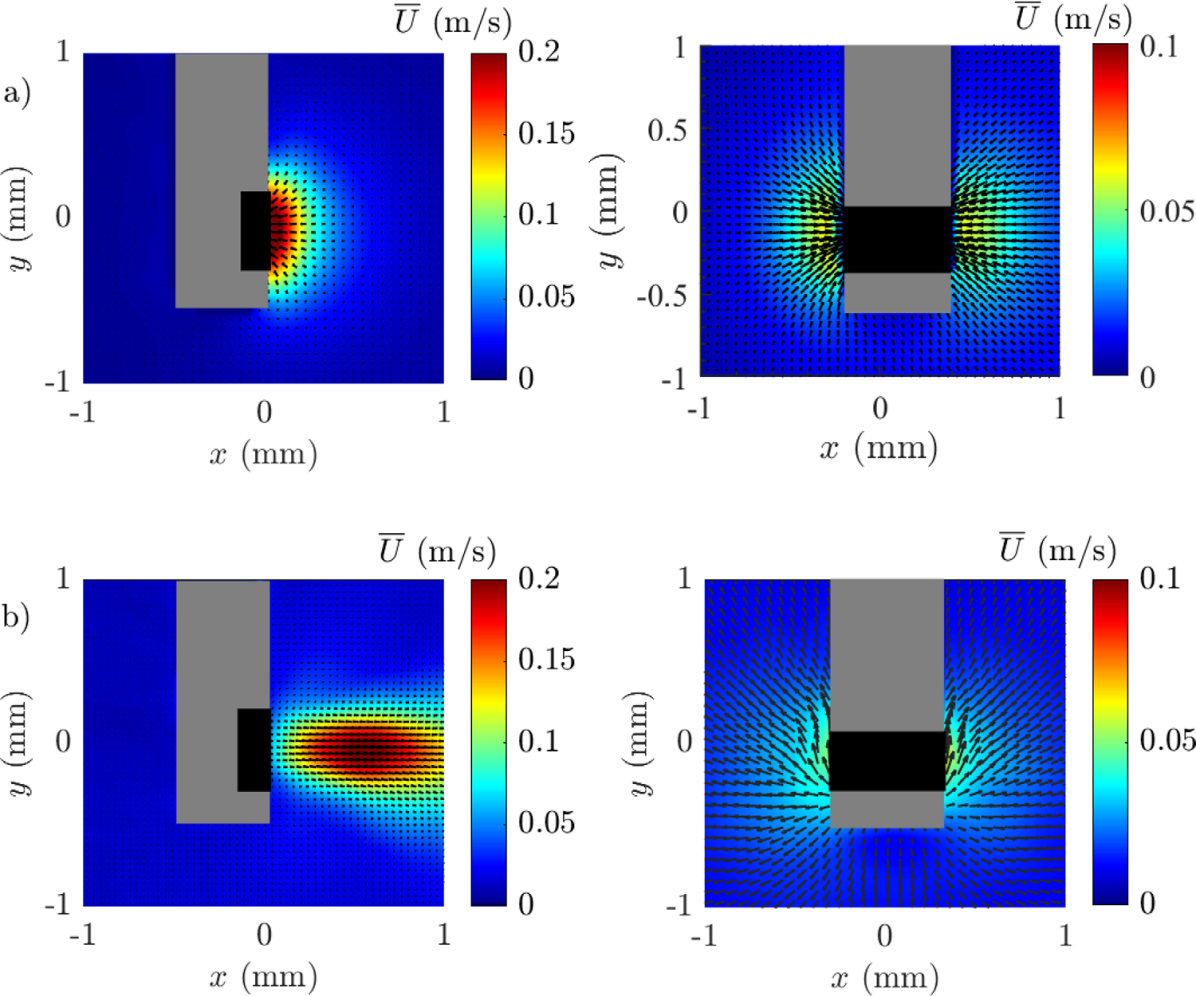
Effect of media on the time-averaged velocity field under matched flow conditions, side (*left*) and front (*right*) views. (**a**) BSS, (**b**) HA-2. Experimental conditions = 25 G, flow controlled aspiration 7 mL/min, and 16,000 CPM.

**Figure 5.**
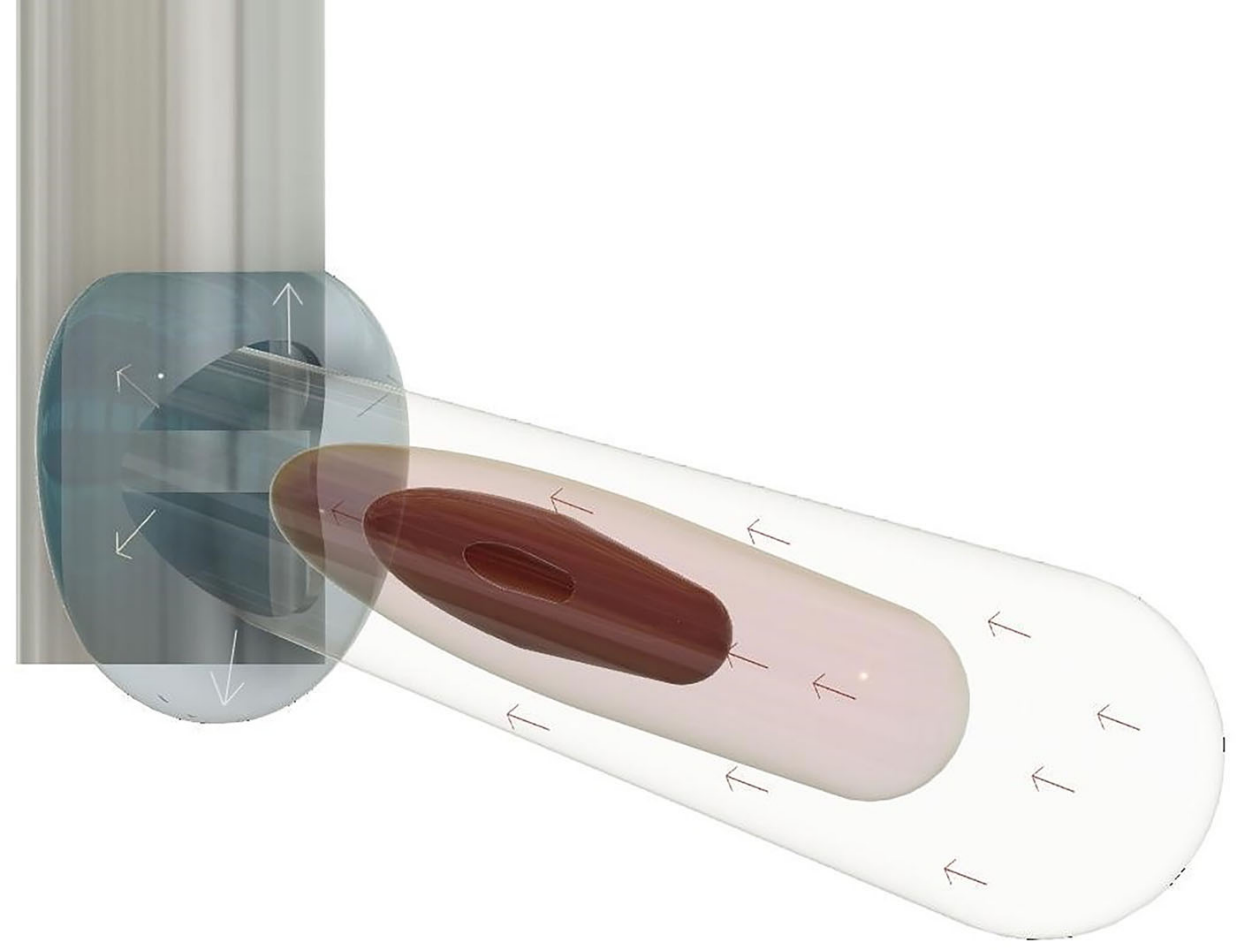
Three dimensional schematic of the FOE around the cutter port. The *arrows* represent the direction of the flow. The *light blue sphere* represents area of low velocity and outflow of the fluid away from the cutter port.

We compared the near velocity fields obtained using HA-2 and BSS under a matched flow rate (7 mL/min) condition with a 25 G cutter operating at 16,000 CPM. Using HA-2 to generate a flow rate of 7 mL/min required a vacuum of 550 mm Hg. The maximum velocity reached approximately 0.20 m/s, with a high-velocity region concentrated near the probe tip (Fig. 4). The flow exhibited a relatively narrow jet extending horizontally, indicating a focused aspiration effect. A representation of the FOE in AVS in a 3D view is provided schematically in Figure 5. Notably, the maximum velocity was reached approximately 0.50 mm eccentric to the port. The jet in AVS maintained a more coherent structure due to the viscoelastic properties of the fluid, with a defined boundary of high velocity. The jet stream effect was confined to the width of the cutter, with only relatively low velocity flow extending out laterally as seen on the port facing view. Interestingly, while all the velocity vectors were directed into the aspiration port with BSS, with AVS velocity vectors were directed away from the port. Using BSS, a vacuum of 100 mm Hg was required to generate a flow of 7 mL/min. The flow field also had a maximum velocity of approximately 0.20 m/s but was evenly spread and symmetrical around the port. The velocity vectors showed a radial sink flow pattern around the cutter, with significant lateral spreading in the lateral plane. Maximum velocity was reached immediately adjacent to the port before declining after a short distance as a function of 1/*x*^2^, with the extent of the high flow being notably more confined than AVS.

**Figure 6.**
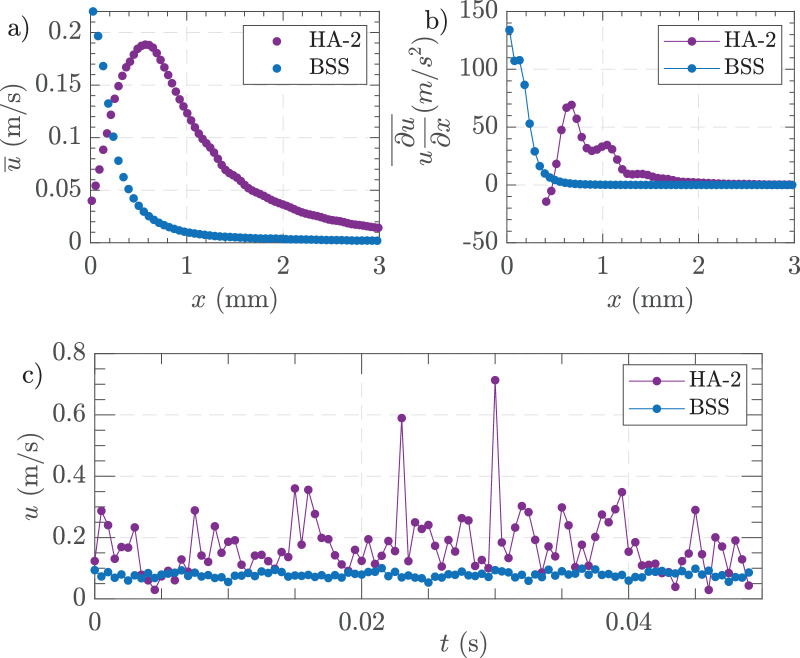
Effect of media on time-averaged horizontal velocity and time-averaged convective acceleration under matched flow conditions. (**a**) Centerline horizontal velocity profiles along the *x* axis for BSS and HA-2. (**b**) Convective acceleration component along the *x* axis for BSS and HA-2, (**c**) instantaneous horizontal velocity measured at (*x, y*) = (0.2, 0) mm in BSS and HA-2. Experimental conditions = 25 G, flow-controlled aspiration 7 mL/min, and 16,000 CPM.

Figure 6a illustrates that the centerline horizontal velocity profiles of HA-2 exhibited a higher peak velocity distant from the cutter than BSS, with an eccentric maximum position. Examination of the time-averaged convective acceleration along the x-axis, Figure 6b, showed that HA-2 experienced peak acceleration, 0.50 to 1 mm from the port in contradistinction to BSS, and then declined in a stepwise fashion approaching 0 at about 2 mm from the port. The instantaneous horizontal velocity measured at 0.20 mm away from the cutter port, Figure 6c, indicates that HA-2 had more pronounced fluctuations in velocity over time compared to the relatively stable profile of BSS. In summary, key differences between the two media included the flow direction and extent of influence with HA-2 showing a focused jet flow into the cutter compared to the more diffused and limited FOE in BSS.

#### Influence of Vacuum on the Field of Fluidic Effect in AVS

**Figure 7.**
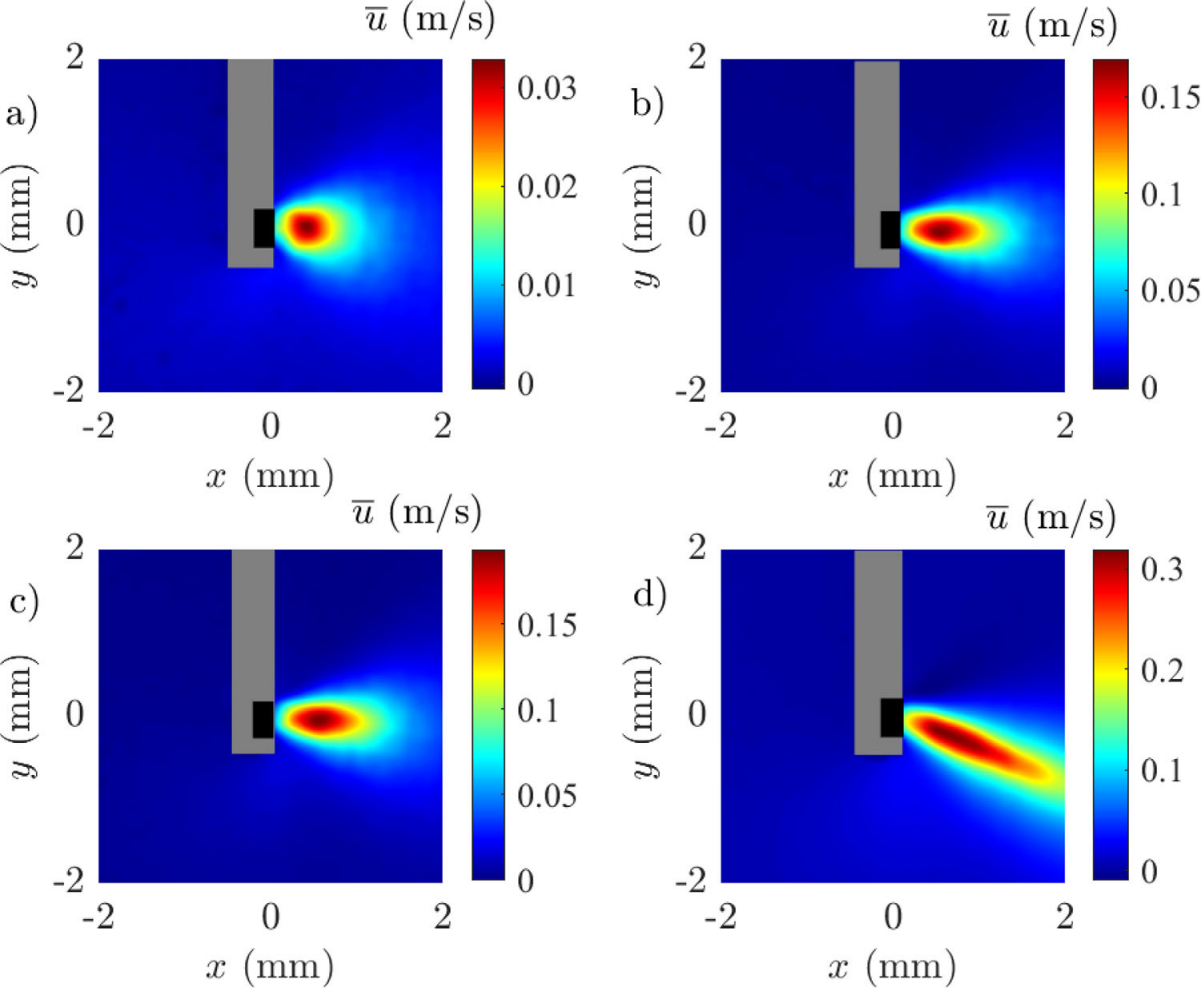
Effect of various vacuum levels on the time-averaged horizontal velocity field at (**a**) 200 mm Hg, (**b**) 400 mm Hg, (**c**) 550 mm Hg, and (**d**) 650 mm Hg. Experimental conditions = 25 G, vacuum controlled aspiration, and 16,000 CPM in HA-2.

**Figure 8.**
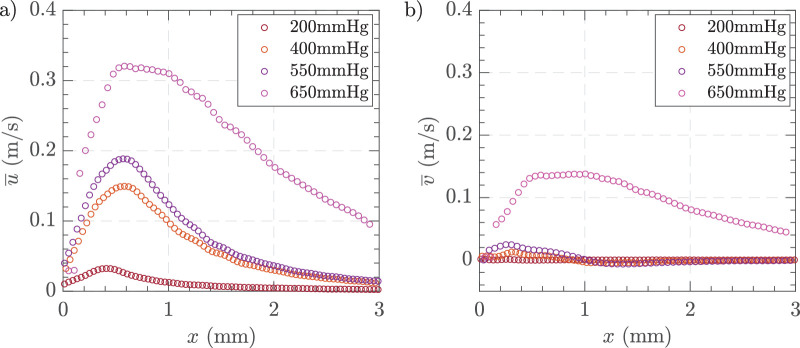
Time-averaged centerline velocity profiles along the *x*-axis for varying vacuum pressures. (**a**) Horizontal velocity component, and (**b**) vertical velocity component. Experimental conditions = 25 G, vacuum controlled aspiration, and 16000 CPM in HA-2.

To investigate the effect of varying vacuum pressures on the flow field around the vitrectomy probe in HA-2, we used a fixed gauge size of 25 G with a cut rate of 16,000 CPM and varied the vacuum levels. Figure 7 shows the time-averaged horizonal velocity fields at vacuum pressures of 200, 400, 500, and 650 mm Hg. Supplementary Figure S4 provides additional data on the time-averaged vertical velocity components of the flow fields for comparison. As vacuum pressure increased from 200 mm Hg to 650 mm Hg, the velocity of the fluid flow increased progressively. At 200 mm Hg, the flow was low with a maximum velocity of 0.05 m/s. As the vacuum increased, the velocity increased becoming a directed jet at 550 mm Hg with velocities up to 0.25 m/s. At the highest pressure of 650 mm Hg, the flow showed a significantly intensified fluidic effect and strong deviation of the jet from the centerline with an angle of 30 degrees to the x-axis. The significant magnitude of the v-component of velocity arises due to the angle generated by the cutter in the Cartesian coordinate system. As the vacuum pressure increased, both the *x* direction (*u*) and *y*-direction (*v*) velocity components showed higher peak velocities (Fig. 8). At 650 mm Hg, the peak horizontal velocity reached approximately 0.30 m/s at approximately 0.60 to 1 mm from the probe, whereas lower pressures had lower peak velocities that peaked at 0.60 mm before declining more rapidly. Similarly, for the vertical component, higher vacuum pressures resulted in higher peak velocities that were sustained further from the probe, indicating greater lateral spreading of the fluid flow.

**Figure 9.**
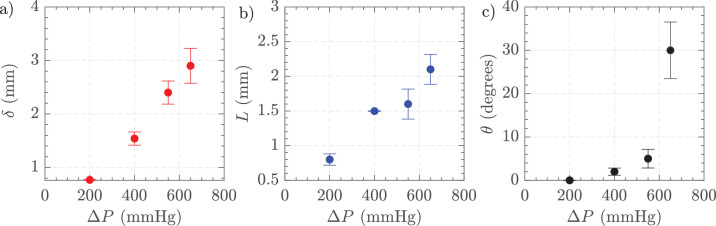
(**a**) Jet thickness, (**b**) jet length, and (**c**) jet angle. Settings = 25 G, vacuum controlled aspiration 200 to 650 mm Hg, and 16,000 CPM in HA-2. The uncertainty bars show the standard deviation of three independent measurements.

Figure 9 depicts the variations in jet characteristics with differing vacuum levels for HA-2 using 25 G and 16,000 CPM. Figure 9a shows the jet thickness (*δ*) indicating an increase in thickness as vacuum increases. Figure 9b presents the jet length (*L*), which also extends with higher vacuum levels. Figure 9c illustrates the jet angle (*θ*), demonstrating a higher deviating angle at higher vacuum pressures. These results highlight the dependence of jet properties on vacuum settings, with all three parameters —thickness, length, and angle— increasing as the vacuum level rises.

### Influence of Cut Rate on the Field of Fluidic Effect in AVS

**Figure 10.**
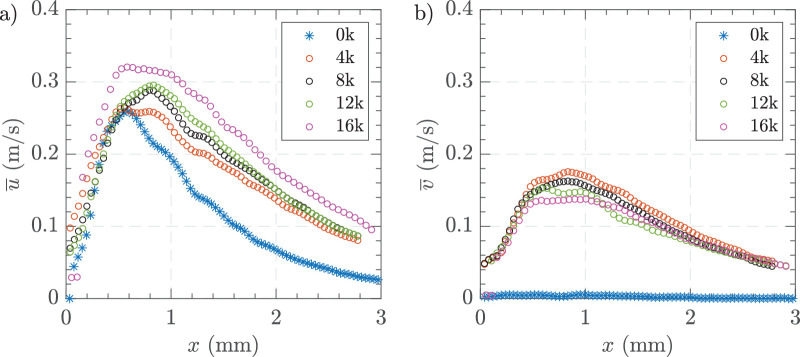
Time-averaged centerline velocity profiles along the *x*-axis for different cutting rates. (**a**) Horizontal velocity component, and (**b**) vertical velocity component. Experimental conditions = 25 G, vacuum controlled aspiration of 650 mm Hg, and 0 to 16,000 CPM in HA-2.

The velocity in Figure 10 illustrates the influence of varying cut rates on the time-averaged centerline velocity around the vitrectomy probe in HA-2. The cut rates ranged from 0 to 16,000 CPM. As the cut rate increased up to 16,000 CPM, the peak velocities for the *x*-direction (*u*) increased, however, in the y-direction (*v*), it decreased, and their peak locations shifted further from the probe tip as compared to the case with the cutter switched off (0 CPM). In the x-direction, the peak velocity at the highest cut rate reached approximately 0.32 m/s around 1 mm from the probe. In the y-direction, the peak velocity at 4000 CPM reached approximately 0.18 m/s. The time-averaged vertical velocity with the cutter action switched off was negligible when compared with cases when the 223 cutting action was on, even in the absence of a deflected jet due to higher applied vacuum.

**Figure 11.**
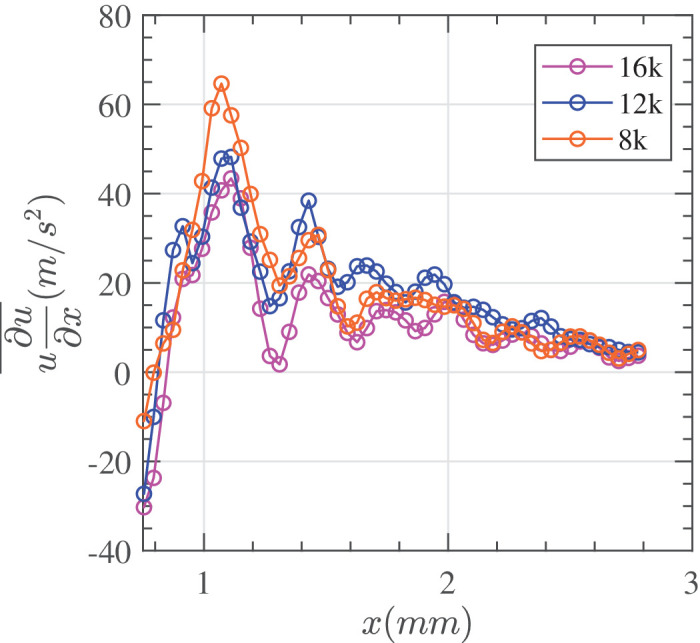
Time-averaged convective acceleration along the *x* axis for 8000, 12,000, and 16,000 CPM with distance from the cutter port. Experimental conditions = 25 G, vacuum controlled aspiration of 650 mm Hg in HA-2.

Figure 11 shows the time-averaged convective acceleration $\bar{u\frac{\partial u}{\partial x}}$ plotted against horizontal distance from the port for 3 different cut rates. Close to the port the acceleration is higher at lower cut rates, but as the distance from the port increases, all three cut rates decrease and converge. Fluctuations in convective acceleration are more pronounced closer to the port.

Power spectral density (PSD) analysis on the fluctuating horizontal velocity at 8000, 10,000, and 16,000 CPM showed distinct signals of the cutter frequency at 70 Hz (frequency of the cutter cycle) and 130 Hz (frequency of the cutting action), 100 and 200 Hz, and 130 and 270 Hz, respectively. There were no significant peaks outside of these frequencies (Supplementary Fig. S5).

Supplementary Figure S6 shows the fluctuations in the horizontal velocity components at 3 cut rates. At all cut rates, the fluctuation peaked at 0.50 mm from the port with similar levels. At the higher cut rates (16,000 CPM), the standard deviations for the horizontal velocity component reduced more rapidly away from the port than lower cut rates suggesting a more stable and consistent flow field. These findings suggest that increasing the cut rate can enhance the stability of the fluid flow around the vitrectomy probe, potentially improving the precision and control during surgical procedures. Supplementary Figure S7 shows the influence of cut rate on jet characteristics for HA-2 and 25G. Figure 7a depicts jet thickness (*δ*) where the thickness remains relatively stable across different cut rates. Figure 7b illustrates jet length (*L*), which shows minor variations with changes in the cut rate. Figure 7c presents jet angle (*θ*), indicating an increase from 0 CPM to 4000 CPM then a slight decrease in angle at higher cut rates. These results suggest that the cut rate has a minimal effect on jet thickness and length, with a modest impact on jet angle.

### Influence of Gauge Size

**Figure 12.**
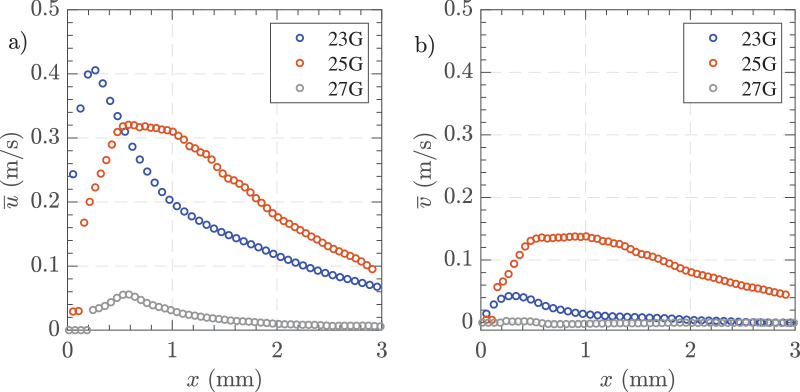
Time-averaged centerline velocity profiles along the *x*-axis for different cutting rates. (**a**) horizontal velocity component, and (**b**) vertical velocity component. Experimental conditions = 23 G, 25 G, and 27 G, vacuum controlled aspiration of 650 mm Hg, and 16,000 CPM in HA-2.

Under matched vacuum conditions, Figure 12, both the larger gauge probes (23 G and 25 G) generated higher peak velocities, whereas the 27 G produced low peak velocities. Specifically, the 23 G probe showed the highest peak velocity near the probe tip at approximately 0.40 m/s, followed by the 25 G probe. Interestingly, the 25 G probe had its peak velocity farther from the probe port, with a plateau of peak flow between 0.50 and 1 mm. In the y-direction, the 25 G probe had the highest peak velocity at approximately 0.14 m/s, which sustained longer than other gauges, consistent with its deviated angle.

**Figure 13.**
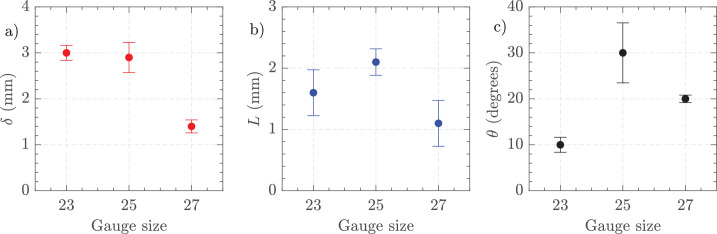
(**a**) Jet thickness, (**b**) jet length, and (**c**) jet angle. Settings = 23 G, 25 G, and 27 G, vacuum controlled aspiration 650 mm Hg, and 16,000 CPM in HA-2. The standard deviation of three measurements is shown at each point.

Figure 13 illustrates the effects of gauge size on jet characteristics for HA-2. Figure 13a shows jet thickness, which was similar for 23 G and 25 G, then reducing for 27 G. Figure 13b displays jet length, showing again a similar length for 23 G and 25 G before reducing for 27 G. Figure 13c presents jet angle, showing a less pronounced angle for 23 G and 27 G but an acute angulation with 25 G as found in the variable vacuum experiments.

**Figure 14.**
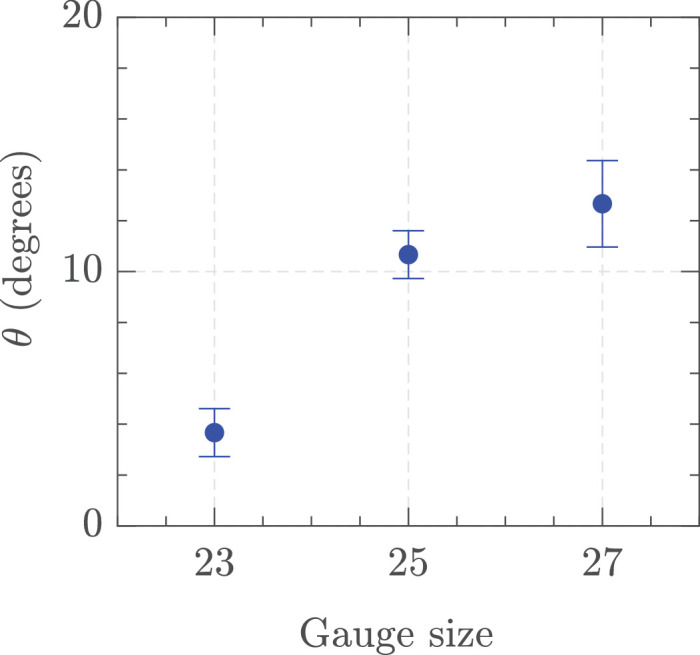
Jet angle. Settings = 23 G, 25 G, and 27 G, vacuum controlled aspiration 650 mm Hg, and 16k CPM in HA-2 in the big tank. The standard deviation of three measurements is shown at each point.

PSD analysis in the two different test chamber sizes showed a similar pattern dominated by the blade and cutting frequencies as before, however, there were some differences with a few additional peaks not corresponding to the above frequencies seen in the smaller tank (Supplementary Fig. S8). We investigated the effect of test chamber size on jet angle as we were suspicious that the wall effects may have affected it. Figure 14 illustrates the jet angles in a larger 100 × 100 × 100 mm test chamber. The angle decreased considerably compared to the smaller test chamber, but there was still a slight increase in angle as the gauge increased.

### Influence of Concentration

**Figure 15.**
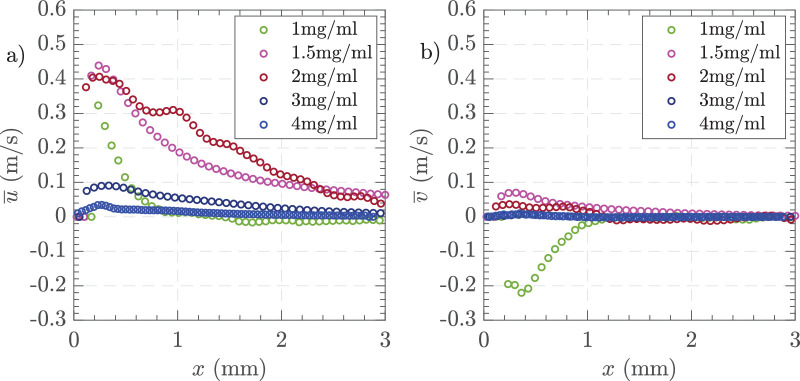
Time-averaged centerline velocity profiles along the *x*-axis for different AVS concentrations. (**a**) Horizontal velocity component, and (**b**) vertical velocity component. Experimental conditions = 23 G, vacuum controlled aspiration of 650 mm Hg, and 16,000 CPM in HA-1, HA-1.5, HA-2, HA-3, and HA-4 in the small tank.

**Figure 16.**
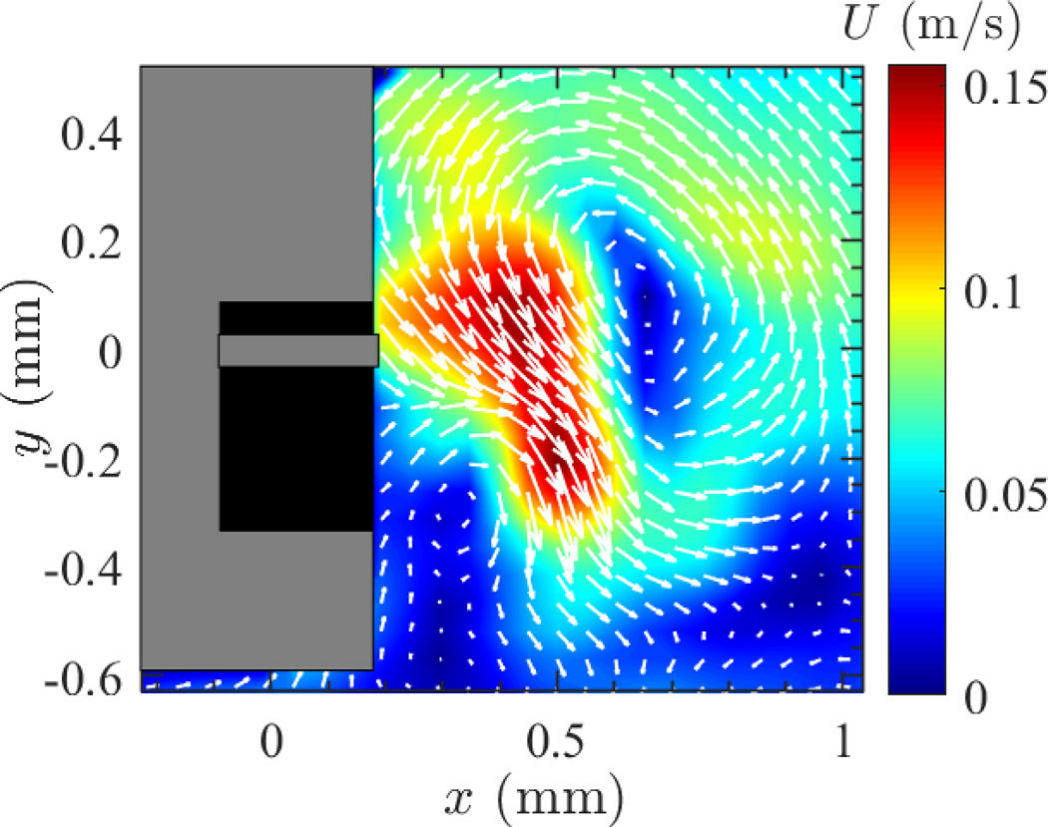
Instantaneous velocity magnitude field with vortices present around the cutter probe. Experimental conditions = 25 G, vacuum controlled aspiration of 650 mm Hg, and 16,000 CPM in HA-4 in the small tank. The *arrows* show the direction of the flow.

Figure 15 shows the time-averaged centerline velocities for different viscosity AVS solutions using 23 G and 16,000 CPM. Figure 15a HA-1 resulted in high velocities immediately adjacent to the probe tip before sharply declining, like the field observed with BSS. As the viscosity increased the peak velocity in the *x* axis moved further away from the port. HA-1, HA-1.5, and HA-2 showed the highest peak horizontal velocities, reaching up to 0.40 m/s near the probe tip. The horizontal velocities for HA-2 showed a more sustained trend with HA-1.5 displaying an intermediate result in between the two. HA-3 and HA-4 had low but sustained horizontal velocity profiles. In the vertical component of velocity, HA-1 showed significant initial negative velocities, indicating strong upward flow near the probe tip that stabilized at around 1 mm, whereas HA-2 and HA-4 displayed smaller variations with velocities close to zero and mainly positive. Visualization of instantaneous horizontal velocities adjacent to the cutter port (Supplementary Fig. S9), showed a higher level of fluctuations for HA-2 than for HA-3. However, with HA-3 the instantaneous velocity dropped to zero periodically, with no horizontal movement. Figure 16 illustrates the formation of a vortex near the vitrectomy cutter port in HA-4, highlighting the fluid dynamics during vitreous aspiration. The high-velocity region near the port suggests a strong shear-driven flow, probably influenced by the cutter duty cycle acting as a piston moving through a cylinder, as was demonstrated by Allen et al. (2000)^21^ and the aspiration rate. The surrounding vortex structure indicates recirculating flow, which may impact retinal traction and vitreous removal efficiency.

**Figure 17.**
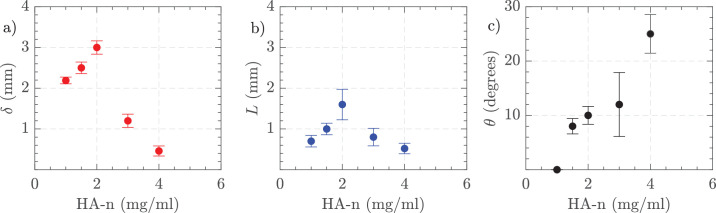
(**a**) Jet thickness, (**b**) jet length, and (**c**) jet angle. Settings = 23 G, vacuum controlled 650 mm Hg, and 16,000 CPM in HA-1, HA-1.5, HA-2, HA-3, and HA-4 in the small tank. The standard deviation of three measurements is shown at each point.

PSD analysis revealed the frequency of the fluctuations with HA-3 to be 133.3 Hz and then several harmonics, for example, 266.6 Hz consistent with the 16,000 CPM cutting rate (Supplementary Fig. S10). PSD analysis in different AVS showed similar patterns and also similar to those observed with BSS. The fluctuation slowly decreased with distance away from the port suggesting a dampening effect. At a point 2 mm away from the cutter port, the values were 0.02 ± 0.02 m/s compared to 0.40 ± 0.40 m/s 0.2 mm away from the port. In Figure 17, we show the impact of AVS concentrations on the FOE produced by aspiration. Figure 17a shows jet thickness, which increased from HA-1 to HA-2, and then sharply decreased as AVS concentration increased to 3 and 4 mg/mL. Figure 17b presents jet length, showing a similar increase and then rapid reduction in length with higher AVS concentrations. Figure 17c displays the jet angle, revealing an increase in angle as the AVS concentration rose. These results suggested that higher AVS concentrations lead to thinner, shorter jets with larger angles, highlighting the influence of AVS concentration on jet properties.

**Figure 18.**
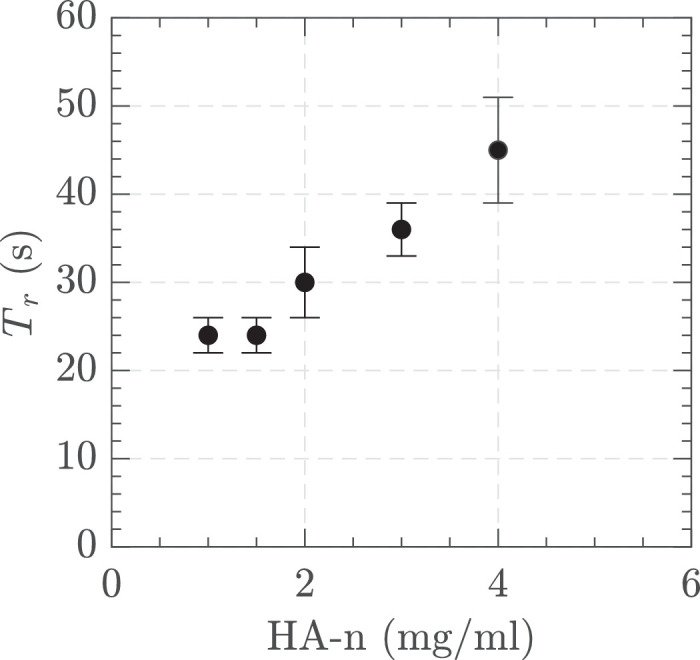
Rise time for the maximum vacuum depending on the concentration of the AVS. Settings = 23 G, vacuum controlled 650 mm Hg, and 16,000 CPM in HA-1, HA-1.5, HA-2, HA-3, and HA-4 in the small tank. The standard deviation of three measurements is shown at each point.

We further investigated the effect of AVS concentration on the time taken to achieve steady state velocity, which we termed ‘rise time’. Each experiment included using a timer to evaluate how long it takes for the machine to reach a steady flow rate. The rise time increased progressively with the AVS concentration. Figure 18 shows as the concentration of AVS increased from 1 mg/ml to 4 mg/ml, the rise time increased from around 25 seconds to just over 45 seconds.

## Discussion

Using PIV and an AVS rheologically matched to liquid component of middle aged human vitreous, we studied the influence of four different surgically relevant parameters on the FOE around a dual action cutting vitrectomy probe. We found that the probe induced a focused but elongated jet flow into the port in AVS as compared to the symmetrical compact sink flow in BSS. Time-averaged flow rates and velocities were lower than BSS as anticipated, but there were also several unexpected findings. The peak time-averaged velocity in AVS was 0.50 to 1 mm away from the port opening and had a broad peak. This peak became broader, with a wider and more elongated jet stream shape with higher vacuum. Furthermore, higher vacuums induced greater vertical velocity components and the jet stream deviated inferiorly away from the port with the highest vacuum levels. Cut rate, in contrast to the situation in BSS increased time-averaged peak velocities of flow, with a variable effect on the jet stream angle and also reduced flow fluctuation. A narrower gauge reduced the flow, as would be expected, but it also affected the shape of the FOE and altered its angle. As the amount of HA in the AVS increased from 1 to 2 mg/mL, the FOE changed from BSS-like to the more elongated profiles described above, with increasing jet length, width, and angulation. Our study thus provides a detailed analysis of the impact of an artificial vitreous media on the field of fluidic effect observed during vitrectomy, offering insights into how it differs from BSS and might interact to influence surgical outcomes. It enhances our understanding of fluid dynamics in vitrectomy, guiding the optimization of surgical techniques and improving patient safety.

The use of AVS has several advantages compared with egg albumen and porcine vitreous, which have previously been used, as the medium rheology is homogenous, consistent between batches and can be tested. This ensures repeatability of testing and also means the rheological properties can be tuned according to the experimental purposes. In our investigation, we used differing concentrations of HA in deionized water to formulate the AVS. Agar has also previously been added to AVS to mimic the fibrillary component of vitreous but we omitted this to maximize the transparency of the AVS for the PIV measurements.^12^ We aimed to mimic human vitreous as much as possible both rheologically and in terms of bulk flow rates and so compared our AVS to human vitreous, as tested by Tram et al.^14^ The rheological properties of human vitreous from relatively young donors were somewhere between our HA-2 and HA-4 solutions but chose to use HA-2 aiming to mimic more aged synergetic vitreous as being more relevant for most vitreo-retinal patients. This also broadly the matched-flow rates achieved using human vitreous in other studies and like those produced by the S3-4 solutions, as used by Nepita et al.,^12^ which included agar, see Supplementary Figures 1a and 1b. Our results validate and add to the findings of Nepita et al.^12^ and Stocchino et al.^9^ They showed that for AVS formulations mimicking aged human vitreous (moduli 1–2 Pa at 1 Hz; viscosities 0.2–0.6 Pa·s at 10² s*^−^*^1^) the flow rose with the vacuum but plateaued at higher cut rates and dual-blade (DB) probes outperformed single-bladed (SB) ones, yielding higher flow and lower fluctuations across all gauges. Our EVA Nexus data concurs with these findings and showed that: (a) AVS flow rates (HA-2, HA-3, and HA-4) were much lower than BSS under identical vacuum and cut-rate settings; (b) DB probes give 20% to 30% higher flow than SB cutters at equal pressure and cut rate; and (c) increasing the cut rate from 4000 to 16,000 CPM reduced the velocity fluctuations and shifted the peak FOE region 0.5 to 1 mm farther from the port. By comparing our FOE images in HA-2 across 23 G, 25 G, and 27 G DB probes to the Nepita et al. study, we confirm similar scaling of curves and jet penetration depths. There are minor quantitative differences (0.02–0.05 m/s lower peak velocities at 650 mm Hg/16,000 CPM) possibly reflecting EVA Nexus's updated pump logic and tubing compliance. Beyond these confirmations, our study contributes three novel insights. First, our high-resolution PIV captured two-dimensional velocity and acceleration fields in the FOE, allowing us to quantify jet thickness, length, and angular deviation, which bulk flow data cannot resolve. Second, our measurement of “rise time” (time to reach steady aspiration) across AVS concentrations revealed viscosity-dependent delays in flow initiation, important for surgical understanding at the start of vitrectomy or during changes in vitreous viscosity. Third, with our broader vacuum and cut-rate experiments we were able to show cut-rate-driven jet narrowing, vacuum-induced FOE broadening and tilting, and the shift from symmetric sink flow in BSS to elongated viscoelastic jets in AVS with trends consistent across gauge sizes.

Romano et al.^4^ also conducted a study comparing the fluidics of SB and DB guillotine vitrectomy probes in BSS and AVS. Although we could not directly compare our rheological measurements to the AVS used by Romano et al.^4^ as the testing conditions were dissimilar, the bulk flow rates were similar to those they observed. Many of our findings also align with theirs in that the flow characteristics significantly differ between Newtonian (BSS) and viscoelastic fluids (AVS) with dual action cutting. They found that flow rates in AVS did not increase with the cut rate whereas several others have found that they do.^3^^,^^8^^,^^22^^,^^23^ Consistent with this and using our high resolution PIV set up we found that the FOE changed with the cut rate, with increasing horizontal velocity (toward the port), whereas reducing in the vertical velocity as the cut rate rose. It has been hypothesized that a reduction in viscosity as the cut rates increase, could explain this effect, although this is disputed.^18^^,^^24^ We also found that the extent of maximum velocity in the FOE increased with the highest cut rate whereas fluctuations in the flow reduced, which could also contribute to the increased flow rates observed. Romano et al.^4^ also noted that time-averaged combined acceleration (sum of the temporal acceleration and the convective acceleration) reduced with the rising cut rate, and we also observed the same effect. Fluid acceleration is a critical parameter due to its correlation with vitreoretinal traction and potential retinal damage. Our experiments confirm the surgical safety of a higher cut rate with reduced flow fluctuation and acceleration but also highlight that flow is increased and the zone of high velocity elongated in AVS which is important for surgical understanding.

The FOE, with its jet flow profile, elongated and widened with increasing vacuum. Unexpectedly, observation of the port facing view showed that all the surrounding velocity vectors were directed away from the port. The translational relevance of this is that removing vitreous within the vitreous base area with the cutter port rotated sideways to the retina should have a low risk of creating iatrogenic retinal breaks. The paradoxical flow directions are a consequence of the non-Newtonian viscoelastic media and are also responsible for the apparent gap in flow between the jet stream effect and the port. In this area, the flow direction will be curved away from the horizontal axis in which we analyzed the PIV. That further can be explained by a sharp increase of first normal stress difference under such shearing conditions. Non-Newtonian fluids can generate recirculating vortices even with a flat plate because of the additional extensional viscosity.^25^^,^^26^ Interestingly, we found that higher vacuum also resulted in a deviation of the jet direction inferiorly away from the shaft, of key relevance in a surgical setting where knowledge of the flow field could optimize surgical strategies and reduce the risk of iatrogenic retinal injury. The effect was greater with 25 G with an angulation of approximately 30 degrees. Other authors have also described deviations of the flow direction in a variety of scenarios. Romano described similar findings to us with deviation in the flow away from the shaft in AVS. The effect was more consistent using dual action cutting and increased with increasing cut rate but the effect was not measured. Rossi et al. showed images of the flow deviating in the angle with egg albumin with angulations both proximally towards the shaft^2^ and distally away from the shaft^5^ in different publications and postulated that these were related to the orientation of vitreous fibrils. Inoue et al. noted angular flow in BSS proximally toward the shaft with beveled tip probes, particularly 27 G but not flat tipped ones.^22^ They also noted that the effect in BSS was greater with dual action probes than single action but noted a paradoxical different direction of deviation in 27 G as compared to 25 G.^27^ They commented that the flow dynamics of porcine vitreous gel were complex to analyze and they found it difficult to evaluate the mean flow direction in the vitreous. We did not find any angulation to the mean flow with BSS in flat ended dual cutting action probes and neither have others.^2^^,^^4^^,^^5^ The AVS used in our experiments does not have a fibrillary component ruling out that the effect is related to this. We investigated whether the effect could be related to a wall effect and did find some evidence to suggest this was the case with a reduction in angle as the test chamber increased in dimension from 45 cm^3^ to 1000 cm^3^. Romano et al. and Inoue et al. used small cubical experimental chambers with a volume of 27 cm^3^, which may explain some of the effects previously found. However, it further raises a question of standardization of the experimental setups and fluids used for better benchmarking and comparability between research groups. The finding suggests that the angle of flow changes with proximity to a surface and deserves further investigation. In fluid dynamics, walls influence flow by creating boundary layers where velocity transitions from zero to free stream speed, causing flow separation at curves and obstacles that leads to turbulence and altered drag. Walls generate pressure gradients that affect flow direction and velocity profiles, and, in confined spaces, interactions with walls can change effective viscosity, particularly in non-Newtonian fluids. Indeed, within an eye where the vitrectomy probe approaches the retina far closer than used in these experiments flow directions may deviate acutely from the orthogonal.

The HA concentration had an interesting effect on the FOE above that of purely reducing flow as might be expected from the viscosity increase. For a set gauge, the FOE gradually transitioned from a symmetrical classic sink flow as seen with BSS to a jet flow as the HA concentration increased. Further increase in AVS concentration led to markedly and relatively sudden reduction in velocity and flow, with a constricted FOE. The effect of the AVS concentration will be directly related to the gauge used. We chose to use 23 G with a vacuum of 650 mm Hg for investigating the effect of AVS concentration. Based on the physical limits of the vacuum, smaller gauges will result in lower flows at the same concentrations and reach this point of sudden flow reductions at lower AVS concentrations. Flow also became deflected away from the port with increasing AVS concentration due to vortex formation. As the HA level increased further and at a level defined by the gauge, the FOE contracted toward the port maintaining its deviation. In clinical scenarios with varying zones of vitreous properties, the same will occur with the FOE changing in spatial extent and angle according to media encountered, explaining some of the variable FOE observed. The residual angular deviation observed in the large chamber may be related to the viscoelastic properties of the AVS. As aspiration occurs up the probe, this will induce a downward vector to the flow.

Romano et al. noted the presence of peaks in the power spectral density plots which did not correspond to the cutting rate and which were not present in BSS. They postulated that they were related to resonant effects and might vary by cutter gauge and the AVS used. Rossi et al.^28^ also noted possible resonant effects in BSS with higher fluctuations in flow at 4000 cuts per minute than higher or lower cutter frequencies. We did not note any clear effects consistent with resonance, but we did note that the amplitude of the PSD varied by the AVS used. We also noted that the PSD plots in smaller test chambers showed increased amplitudes and low peaks that did not correspond to the cutting or blade cycle frequencies. It is possible that differences in the experimental design between studies could explain these variable findings. It does also raise the possibility, however, that vitreous cavities with volumes, typically of 4 to 6 cm^3^ may induce resonant effects dependent on vitreous viscosity.

Missel et al.^29^ modeled vitreous removal using computational fluid dynamics and finite element analysis in a virtual 8 mm × 3.3 mm cylinder. Their model was useful in terms of parameter optimization but simplifications of vitreous rheology and geometry were used, limiting the precise portrayal of FOE attributes. In contrast, our experiments revealed that AVS's viscoelasticity turned the symmetric flow seen with BSS into elongated, vortex-laden jets under high vacuum. We found that the vacuum lengthened and tilted the jets, higher cut rates stabilized the flow and reduced fluctuations, and both gauge size and HA concentration notably reshaped flow profiles. Together, all these studies confirm that the cut rate enhances safety (reducing traction/instability), the vacuum demands caution (amplifying jet deviation), and rheology governs flow, underscoring the need for surgeons to dynamically balance these factors near the retina.

We accept that our study has several limitations that need to be addressed in future studies. We showed that the proximity of the test chamber wall can influence the FOE which requires further study. This will require further experimentation and potentially simulation to assess the effect of range of relevant surgically important geometries. The AVS used did not have a fibrillary component, limiting our ability to investigate the effect of elastic recoil and fiber orientation on the FOE. Omitting a mimic of vitreous fibers might have altered the FOE from reality. In the absence of collagen fibrils, the jet deflection (up to 30 degrees at high vacuum) we observed stems from viscoelastic and wall effects, whereas real vitreous fibers impose additional anisotropic resistance, directing jets along paths of least obstruction.^5^^,^^30^ In AVS, vortices dissipate rapidly without a fiber network, whereas in vivo, collagen bundles may have variable effects, sometimes sustaining vortices, storing elastic energy, and potentially amplifying tractional effects, and other times having dampening effects depending on the local rheological conditions.^30^^,^^31^ Furthermore, we did not include an infusion during the experiments. The orientation and velocity of the infusion fluid with respect to the vitrectomy probe varies widely during in vivo vitrectomy, adding considerable complexity to the fluidics beyond the scope of this initial study. Moreover, we did not vary intraocular pressure which may also affect the flow characteristics involved. Finally, we only studied flow in two planes relative to the vitrectomy probe and 3D methodologies of PIV, with stereoscopic and particle tracking the velocity methodologies may add further insight.

## Conclusions

Using an AVS with similar rheological properties to the fluid component of human vitreous and a high-speed PIV technique, we evaluated the FOE around vitrectomy probes with a range of surgically relevant variables. In our research, we examined the influence of various parameters on fluidic effects, including cut rate, vacuum pressure, AVS concentration, gauge size, and tank size. We found that higher vacuum pressures lead to stronger fluidic effects, whereas increased cut rates result in more stable, higher peak velocities. Larger gauges produced longer, thicker jets, with smaller gauges causing a jet angled with respect to the port opening. AVS concentration affected flow behavior up to 3 mg/mL, beyond which flows became limited, with impulsively started miniature vortices observed close to the cutter port due to blade action in the more viscous media. Additionally, tank size significantly impacted the results due to wall effects. Overall, our findings provide valuable insights into the fluid dynamics during vitrectomy, helping to optimize surgical techniques and improve patient outcomes. Further research into the influence of different vitreous substitutes and surgical parameters is crucial to enhance our understanding and refine vitrectomy procedures.

## Supplementary Material

Supplement 1
