# Supplementary material for: The Influence of Fluids With Varying Rheological Properties on the Field of Fluidic Effect During Vitrectomy
Source: Transl Vis Sci Technol. 2025 Aug 20;14(8):26. doi: 10.1167/tvst.14.8.26 (PMC12372945; doi:10.1167/tvst.14.8.26)
Supplement: Supplement 1 [file tvst-14-8-26_s001.pdf]

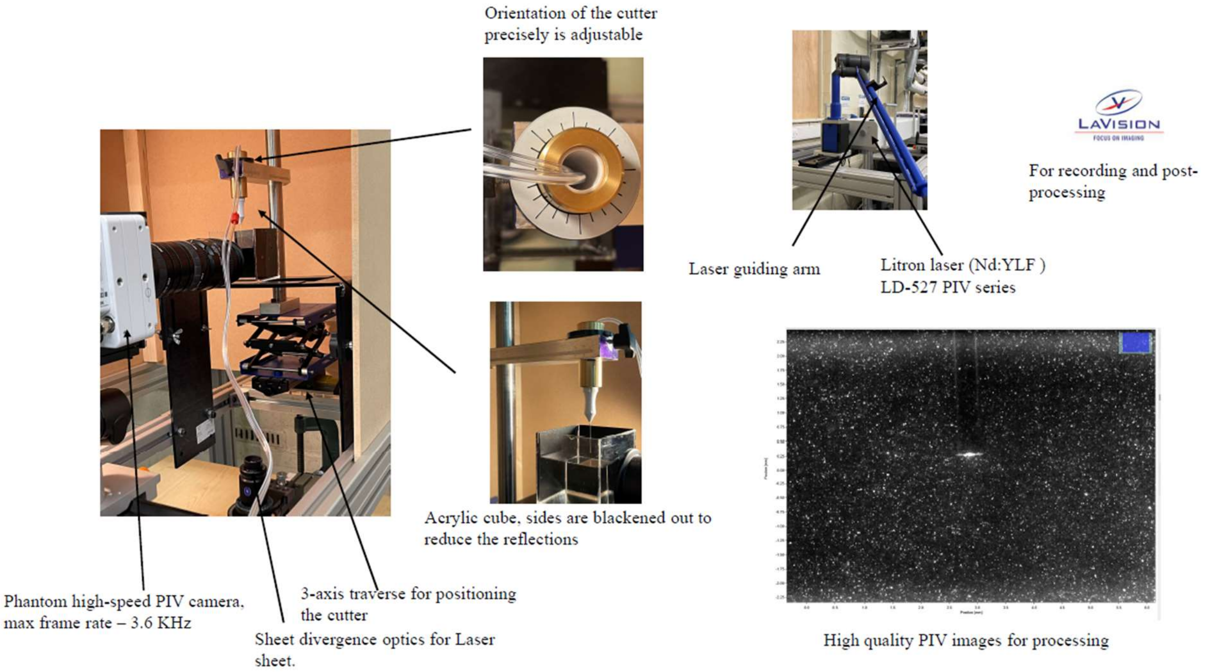

Supplementary Figure 1. Actual bench-top setup, showing (i) the custom holder with adjustment knob for 1° rotation, (ii) the three-axis manual traverse (with micrometre dials indicating 0.1 mm steps in x/z and 1 mm in y), and (iii) the PIV laser guiding arm and fluid tank

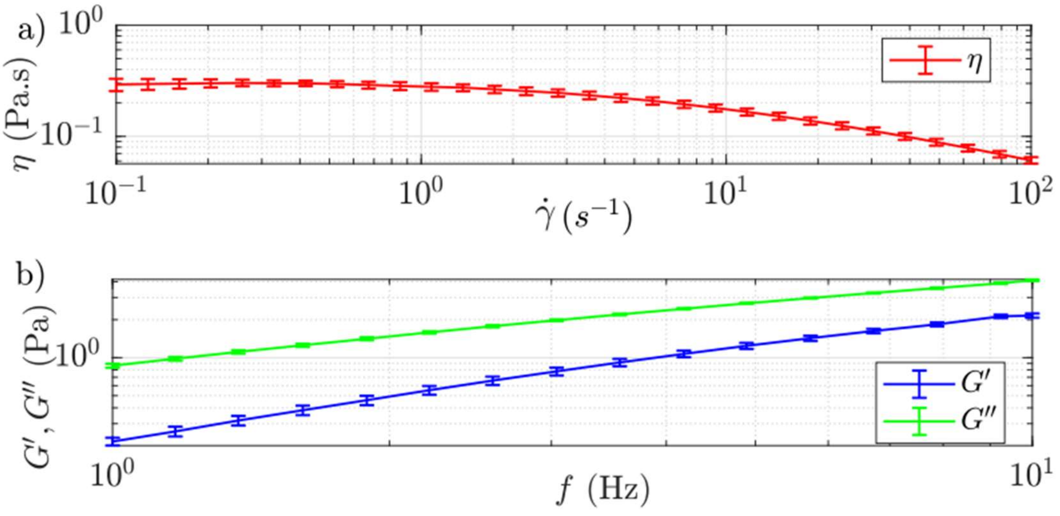

Supplementary Figure 2: Stability testing of HA-2 at 25oC across 5 days. a) Shear viscosity η as a function of shear rate and b) Storage modulus G’ and loss modulus G” as functions of frequency. Uncertainty bars represent the difference between daily

measurements.

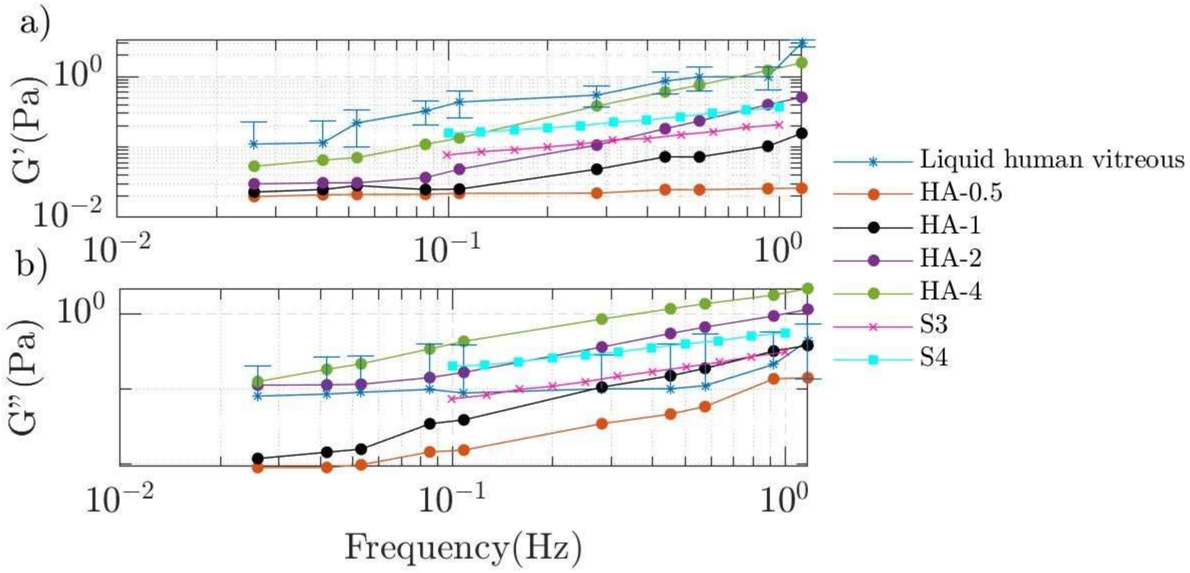

Supplementary Figure 3: Rheological properties of the AVS solutions at 25 °C Oscillatory steady-state tests: a) Storage modulus G' and b) Loss modulus G". Liquid human vitreous refers to data from Tram et al 2018. Human vitreous samples (n = 39, aged 62 ± 15 years). According to the authors, error bars represent 95% confidence intervals for measurements [12]. S3 and S4 refers to solutions produced by Nepita et al 2021. According to authors, the fluids obtained are viscoelastic. They note that hyaluronic acid is a natural component of the real vitreous, which, together with collagen, contributes to the biomechanical properties of the vitreous. On the contrary, Agar powder is not present in the vitreous but it represents a good surrogate for collagen fibrils in terms of elastic properties[11].

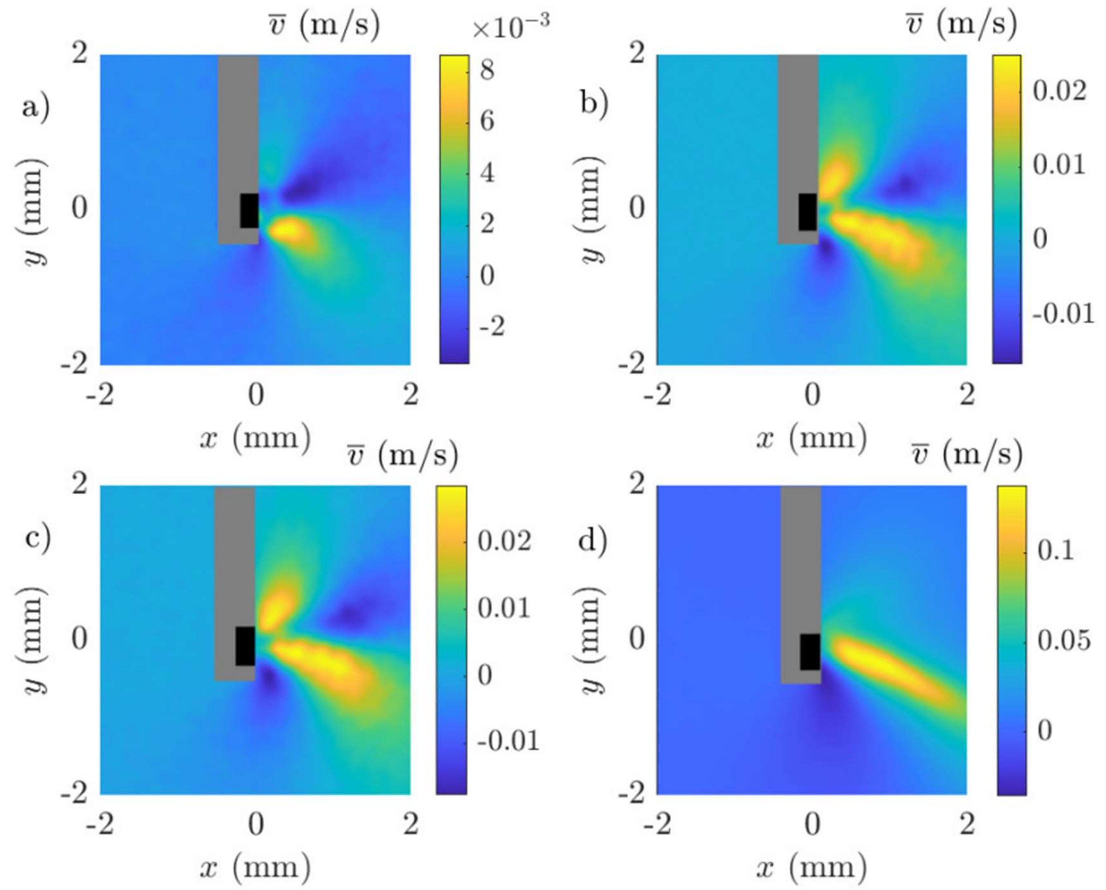

Supplementary Figure 4: Effect of various vacuum levels on the time-averaged vertical velocity field at (a) 200 mmHg, (b) 400mmHg, (c) 550 mmHg, (d) 650 mmHg. Experimental conditions: 25G, vacuum controlled aspiration, 16,000 CPM, in HA-2.

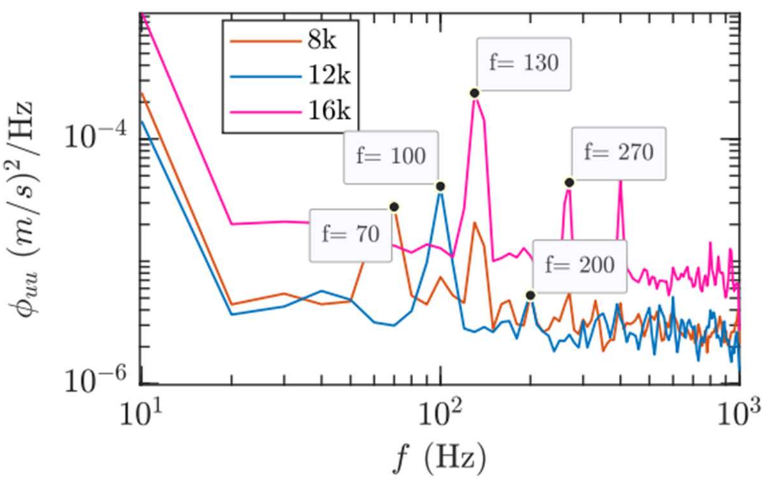

Supplementary Figure 5: Power spectral densities of fluctuating horizontal velocity component at 0.2 mm away from the cutter port along the x - axis. Experimental conditions: 25G, vacuum controlled aspiration of 650 mmHg, 8,000, 12,000 and 16,000 CPM, in HA-2.

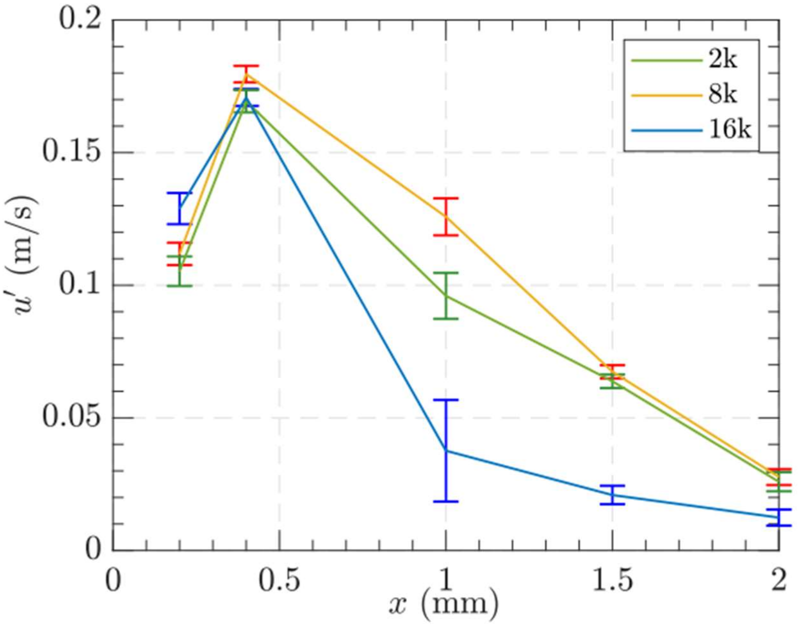

Supplementary Figure 6: Standard deviation for horizontal velocity component fluctuations along x-direction for 2,000, 8,000, and 16,000 CPM with vacuum controlled aspiration of 650 mmHg. The standard deviation of 3 measurements is shown at each point.

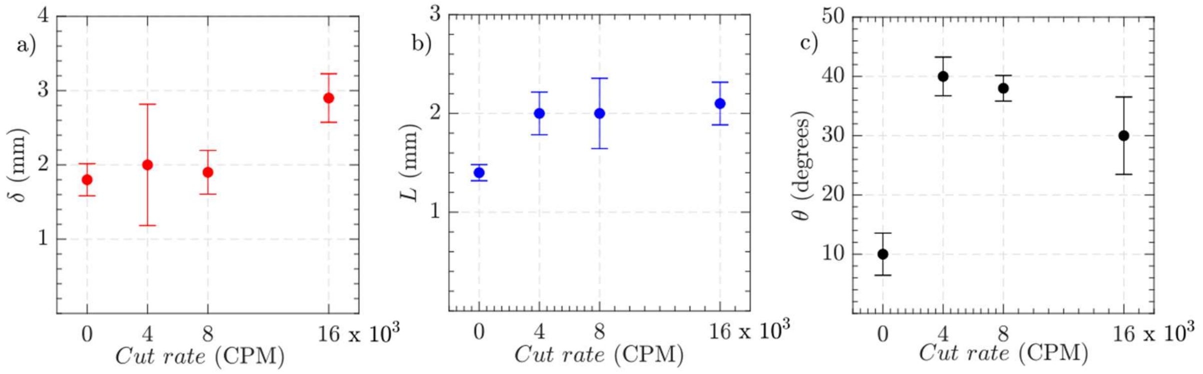

Supplementary Figure 7: (a) Jet thickness, (b) jet length and (c) jet angle; Settings: 25G, vacuum controlled aspiration 650mmHg, 16,000 CPM in HA-2. The standard deviation of 3 measurements is shown at each point.

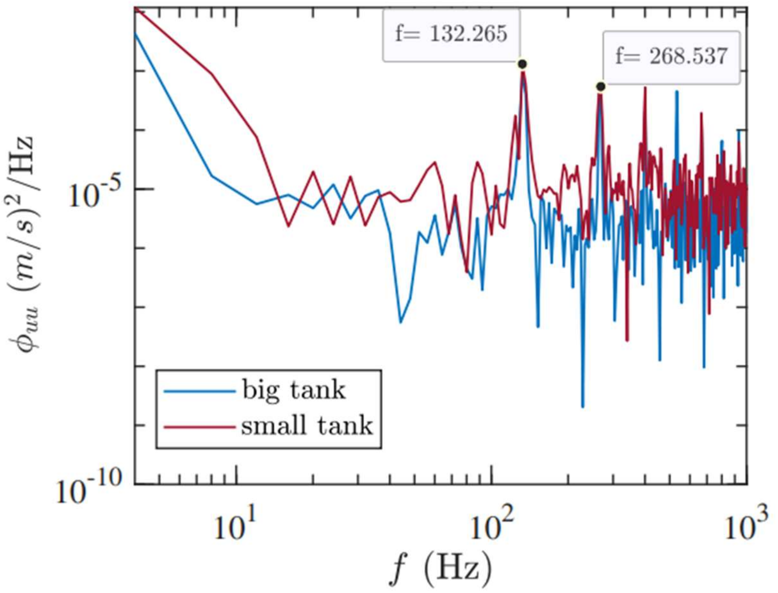

Supplementary Figure 8: Power spectral densities of fluctuating horizontal velocity component. Experimental conditions: 25G, vacuum controlled aspiration of 650 mmHg, 16,000 CPM, in HA-2. Big tank 100x100x100 mm, small tank 35x35x55 mm. Measurement point - 0.2 mm away from the cutter port along x - axis.

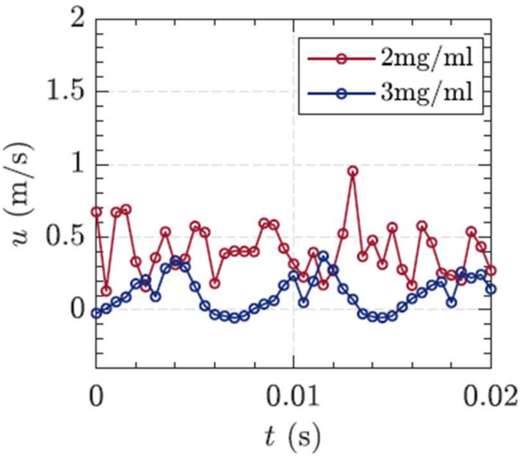

Supplementary Figure 9: Instantaneous horizontal velocities at 0.2 mm away from the cutter port. Experimental conditions: 23G, vacuum controlled aspiration of 650 mmHg, 16,000 CPM, in HA-2 and HA-3 in the small tank.

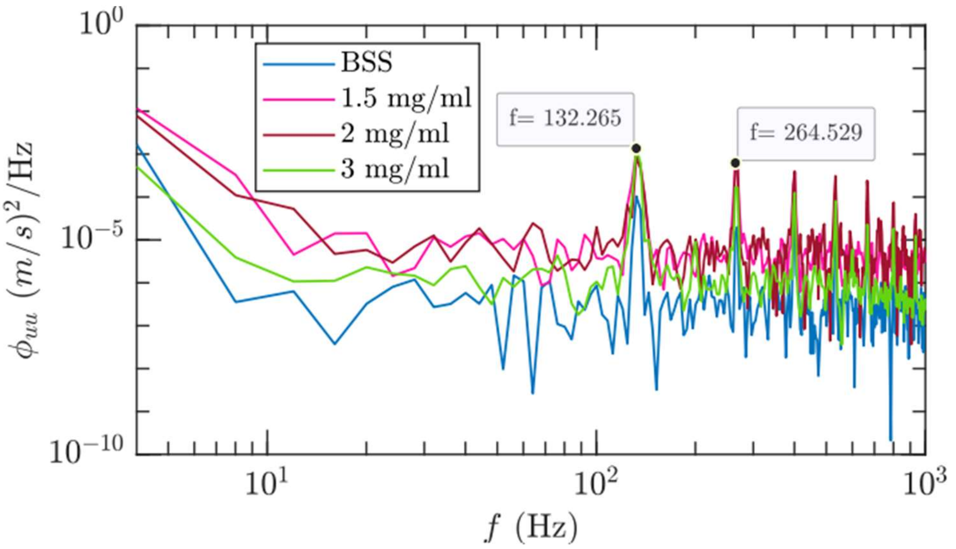

Supplementary Figure 10: Power spectral densities of fluctuating horizontal velocity component. Experimental conditions: 23G, vacuum controlled aspiration of 650 mmHg, 16,000 CPM, in BSS and HA-1.5, HA-2, HA-3 in small tank. Measurement point - 0.2 mm away from the cutter port along x - axis.
